# Integrating Health Care Data in an Informatics for Integrating Biology & the Bedside (i2b2) Model Persisted Through Elasticsearch: Design, Implementation, and Evaluation in a French University Hospital

**DOI:** 10.2196/65753

**Published:** 2025-04-24

**Authors:** Romain Griffier, Fleur Mougin, Vianney Jouhet

**Affiliations:** 1 Service d’Information Médicale, Informatique et Archivistique Médicale (IAM) Pôle de Santé Publique Bordeaux University Hospital Bordeaux France; 2 Team AHeaD Inserm Bordeaux Population Health Research Center, UMR 1219 Bordeaux University Bordeaux France

**Keywords:** clinical data warehouse, health data integration, i2b2, Elasticsearch, medical informatics, data persistence

## Abstract

**Background:**

The volume of digital data in health care is continually growing. In addition to its use in health care, the health data collected can also serve secondary purposes, such as research. In this context, clinical data warehouses (CDWs) provide the infrastructure and organization necessary to enhance the secondary use of health data. Various data models have been proposed for structuring data in a CDW, including the Informatics for Integrating Biology & the Bedside (i2b2) model, which relies on a relational database. However, this persistence approach can lead to performance issues when executing queries on massive data sets.

**Objective:**

This study aims to describe the necessary transformations and their implementation to enable i2b2’s search engine to perform the phenotyping task using data persistence in a NoSQL Elasticsearch database.

**Methods:**

This study compares data persistence in a standard relational database with a NoSQL Elasticsearch database in terms of query response and execution performance (focusing on counting queries based on structured data, numerical data, and free text, including temporal filtering) as well as material resource requirements. Additionally, the data loading and updating processes are described.

**Results:**

We propose adaptations to the i2b2 model to accommodate the specific features of Elasticsearch, particularly its inability to perform joins between different indexes. The implementation was tested and evaluated within the CDW of Bordeaux University Hospital, which contains data on 2.5 million patients and over 3 billion observations. Overall, Elasticsearch achieves shorter query execution times compared with a relational database, with particularly significant performance gains for free-text searches. Additionally, compared with an indexed relational database (including a full-text index), Elasticsearch requires less disk space for storage.

**Conclusions:**

We demonstrate that implementing i2b2 with Elasticsearch is feasible and significantly improves query performance while reducing disk space usage. This implementation is currently in production at Bordeaux University Hospital.

## Introduction

Health data account for approximately 30% of the world’s available data [1]. Moreover, the volume of digital health data is constantly increasing [2]: in 2010, it doubled every 3 years; by 2020, it was estimated to double every 73 days [3].

One source of health data is electronic health records (EHRs). EHRs are defined as a “longitudinal collection of electronic health information about individual patients” [4], generated from 1 or more encounters in any care delivery setting. They include patient demographics, clinical notes, medications, vital signs, medical history, laboratory data, and radiology reports.

Health data, particularly EHR data, may be used for secondary purposes. Secondary use of health data refers to its use for purposes other than those for which they were initially collected [5]. This includes a wide range of areas [6], such as managing health care organization activities, phenotyping [7,8], research [9], epidemiological registers [10], quality and safety of care [11], and epidemiological surveillance [12,13].

One barrier to reusing health data from EHRs is its extreme heterogeneity [14,15]. Two types of heterogeneity are generally distinguished:

- Syntactic heterogeneity: This refers to differences in how information is stored, such as in different formats (eg, databases, text files, or images) or using various data models in specialized software.
- Semantic heterogeneity: This refers to differences in how information is represented. For example, a patient’s natremia may be stored in a structured format using a LOINC (Logical Observation Identifiers Names and Codes) [16] code (eg, “2947-0”), a local terminology code (eg, “BIO:NaSg”), or as free text in a clinical note.

These 2 types of heterogeneity create challenges in the secondary use of health data. Various strategies have been implemented to reduce this heterogeneity, including machine learning and rule-based methods [17] during analysis. A widely adopted approach is to integrate data before analysis by establishing clinical data warehouses (CDWs) [18-22].

From a technical perspective, a CDW is a database dedicated to the secondary use of health data. CDWs are populated through a process called ETL (Extract, Transform, and Load), in which health care data are extracted from various production or replicated medical software databases, transformed (including pseudonymization and standardization), and then loaded into a dedicated database. Beyond being just a database, CDWs must also incorporate key aspects necessary for health data reuse, including governance, ethics, transparency, privacy, and security.

Numerous models for integrating data into CDWs have been proposed [23]. In particular, 2 open data models are widely used worldwide:

- OMOP-CDM (Observational Medical Outcomes Partnership-Common Data Model): Developed through a public-private partnership established in 2008 and led by the Food and Drug Administration [19], OMOP-CDM is a denormalized data model in which clinical data are stored in 1 of 15 specialized fact tables (eg, DRUG_EXPOSURE, MEASUREMENT) within the “Standardized Clinical Data” section of the CDM. All data in these fact tables are linked to the main PERSON table, which represents the patient. Additionally, 12 tables in the “Standardized Vocabulary” section store and manage the vocabulary integrated into OMOP. The current version of OMOP-CDM is 5.4 [24].
- Informatics for Integrating Biology & the Bedside (i2b2): Proposed by Harvard Medical School’s Department of Biomedical Informatics in 2007 [18], the i2b2 platform provides a tool called the i2b2 Web Client for identifying patients based on clinical or biological criteria. A description of this requester is provided in Multimedia Appendix 1. A more detailed explanation of the i2b2 application and its data model is included in the “Materials” section.

In the remainder of this article, we will focus specifically on i2b2.

One of the first steps in the secondary use of health data is conducting feasibility studies [25] and phenotyping [26,27]. Feasibility studies involve counting candidate patients, while phenotyping focuses on identifying patients eligible for studies based on clinical or biological criteria. To achieve these tasks, researchers query CDW data to precisely define the population of interest. Phenotyping queries must meet 2 key criteria: (1) *good recall*, that is, they must capture the population of interest as comprehensively as possible; and (ii) *good precision*, that is, the identified patients must accurately match the search criteria. The development of these queries is an iterative process: the initial counts and record visualizations help refine the query by identifying additional elements to consider. Therefore, query response times must be very fast—on the order of 1 second—to ensure that this iterative process remains feasible and efficient.

In addition, a significant portion of medical information is available in free text [21,28], such as clinical notes, imaging reports, and discharge prescriptions. Within the i2b2 platform, free-text data are extracted from binary documents (eg, .docx, .pdf) and stored in a dedicated field. The search engine enables querying this free-text information using keyword-based exact searches.

It is therefore essential to use data storage systems for CDWs that enable rapid querying and efficient methods for searching free text. Most CDWs rely on a relational database to store data. Relational database management systems (RDBMSs) are well-suited for ensuring data integrity and provide a simple yet flexible query language, SQL. However, the query performance of RDBMSs may be challenged when handling large volumes of data or a high number of users [29-32]. This is because RDBMSs strictly adhere to the ACID (atomicity, consistency, isolation, and durability) properties to ensure data integrity and consistency. Maintaining these properties introduces mechanisms such as resource locking, which can impact query speed. Additionally, while RDBMSs are highly efficient for storing structured data, they are less suited for handling semistructured or unstructured data, such as free text [33,34].

NoSQL databases emerged in the 2000s with the rise of Web 2.0 [35] and have been widely used in big data and real-time applications. Unlike traditional relational databases, NoSQL databases do not store data in a relational manner. A key difference is that NoSQL databases are schema-free, meaning their table structure is not fixed—the structure of stored data may vary between instances [36]. There are different types of NoSQL databases, including key-value databases, document-oriented databases, column-oriented databases, and graph databases [37].

Elasticsearch [38] is a NoSQL database that stores data as JSON documents in a schema-less structure, optimized for full-text search and real-time analytics. It supports horizontal scaling across clusters and uses a query domain-specific language for complex searches, making it well-suited for indexing, querying, and aggregating large volumes of heterogeneous data. Therefore, we considered Elasticsearch a strong candidate for CDW data persistence in the context of i2b2’s search engine for the phenotyping task. This paper aims to present our implementation and evaluate the query performance associated with persisting the main components of the i2b2 data model in an Elasticsearch NoSQL database. A portion of the i2b2 query engine has been reimplemented for performance evaluation, focusing on counting queries based on structured data, numerical data, and free text, including temporal filtering.

## Methods

### Materials

#### Informatics for Integrating Biology & the Bedside (i2b2)

**Figure 1:**
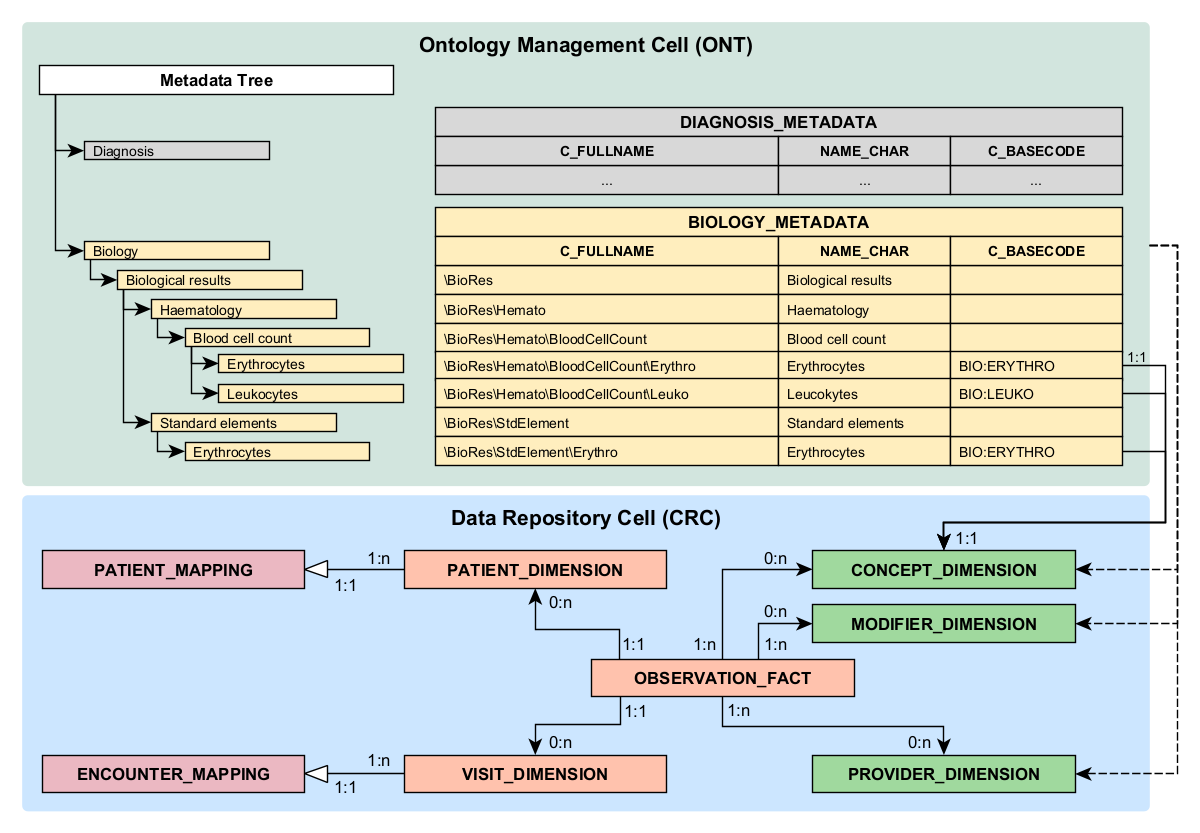
Informatics for Integrating Biology & the Bedside (i2b2): Data Repository Cell (CRC) and Ontology Management Cell (ONT). In the CRC (blue box), tables containing pseudonymized patient data are shown in orange, mapping tables with the integrated source(s) are in red, and metadata dimension tables are in green. In the ONT, metadata are organized into distinct metadata tables (eg, BIOLOGY_METADATA, DIAGNOSIS_METADATA), each containing tuples that represent nodes in a hierarchical tree structure. Each node in the tree corresponds to a tuple in the metadata tables, with its primary key representing the path from the root node to the current node. For example, the primary key of the intermediate node “Blood cell count” is C_FULLNAME = “\BioRes\Hemato\BloodCellCount.” Leaf nodes are associated with a code stored in the C_BASECODE column. Only the tuples in the metadata tables that correspond to leaf nodes are included in the metadata dimension tables. For instance, the tuples for “Leucocytes” and “Erythrocytes” are included in the CONCEPT_DIMENSION table.

i2b2 is an application developed by the Department of Biomedical Informatics at Harvard Medical School in 2007 [18]. It aims to facilitate the integration of heterogeneous clinical health care data for secondary use by providing data persistence storage and offering various services on top of this storage. The application follows a service-based architecture, comprising multiple services (referred to as “cells”). Specifically, the i2b2 application distinguishes the following 2 cells (Figure 1):

- The *data repository cell* (also called the clinical research chart (CRC); blue box in Figure 1), which stores clinical information generated during a patient’s encounter with a health care facility. The CRC is based on a star-schema model [39] composed of the following:
  A section containing *pseudonymized individual clinical data* (shown in orange in Figure 1; the names of RDBMS objects [tables, columns, etc] are written in UPPERCASE and the names of Elasticsearch objects [indexes, fields, etc] are written lowercase.). The individual clinical information integrated into i2b2 is stored as observations in the main fact table, OBSERVATION_FACT. Each observation represents a piece of information about a patient’s health, collected during an interaction with the health care system. This may include structured elements (eg, diagnoses coded using standard terminology, numerical biological results) as well as free-text elements (eg, clinical notes, form data). This table is linked to 2 dimension tables: PATIENT_DIMENSION, which describes patient attributes (eg, gender, date of birth), and VISIT_DIMENSION, which describes encounter details (eg, start and end dates).
  A section describing the permissible values of the lookup columns (the lookup columns correspond to columns containing structured elements used for searching in the OBSERVATION_FACT table. These columns are similar to foreign keys of dimension tables, except that they do not refer to the primary key but to other columns in these dimension tables) in OBSERVATION_FACT (shown in green in Figure 1). Specifically, 2 tables define the concepts used to characterize an observation: CONCEPT_DIMENSION represents the main concept. For example, the CONCEPT_CD “BIO:ERYTHRO” refers to the number of erythrocytes in blood; MODIFIER_DIMENSION represents a secondary concept that modifies the main concept. For example, the MODIFIER_CD “BIO:VALID” refers to a validated result and can modify an observation linked to the CONCEPT_CD “BIO:ERYTHRO,” indicating that the erythrocytes in blood measurement are validated.
  A section containing *mapping tables* that link patient (PATIENT_MAPPING) and encounter (ENCOUNTER_MAPPING) identifiers used in i2b2 to the corresponding identifiers from the source systems integrated into i2b2 (shown in red in Figure 1).
- The *ontology management cell* (also called ONT; green box in Figure 1) enables the integration of metadata from various sources into a hierarchical tree structure. Metadata are stored in separate tables, each corresponding to a root node of the tree (eg, “Biology,” “Diagnosis,” “Procedure”). In these metadata tables, each row represents a node in the hierarchical tree. The primary key (C_FULLNAME) corresponds to the path from the root node to the current node (either an intermediate or a leaf node). This structure allows for multihierarchical access to leaf nodes, meaning a single leaf node can be accessed through multiple paths. For leaf nodes only, the C_BASECODE column contains the code used as a permissible value in one of the lookup columns (eg, CONCEPT_CD) of the OBSERVATION_FACT table.

In addition to providing meaning to the observations stored in the CRC’s OBSERVATION_FACT table, metadata enable the creation of views on these data through various access paths to lookup codes (eg, CONCEPT_CD), which contextualize each observation. Moreover, separating data and metadata into 2 distinct cells allows the loading of pseudonymized individual clinical data to be decoupled from the loading of metadata describing these data. As a result, modifying the metadata in the ONT makes it possible to adjust the view of the data without altering the data itself. This decoupling of data and metadata is one of the key strengths of the i2b2 architecture.

The i2b2 platform includes a patient and encounter identification engine that queries the ONT and CRC components based on clinical or biological criteria. A detailed description of this engine is provided in Multimedia Appendix 1.

The i2b2 data model is designed for data persistence in an RDBMS [40].

#### i2b2 Within the Bordeaux University Hospital

The Bordeaux University Hospital is a public health care facility in Bordeaux, Nouvelle-Aquitaine, France. Each year, a number of distinct patients visit the hospital for inpatient or outpatient care.

Since 2018, the Bordeaux University Hospital has implemented a CDW based on i2b2. The CDW integrates heterogeneous health data from patients who have visited the hospital since 2010. For these patients, data available since 2005 have also been integrated.

Various types of clinical data are integrated into the CDW:

- Structured data coded using standard terminologies, such as diagnoses (coded with the International Statistical Classification of Diseases and Related Health Problems, 10th Revision [ICD-10] [41]) or drug prescription/administration data (coded with the Anatomical Therapeutic Chemical classification [42]).
- Structured data coded using local (or interface) terminologies [43], such as biological results.
- Semistructured data, recorded in care forms. This type of data includes structured or free-text elements contextualized by a strong organizational structure within the forms: questions are grouped into sections, which are further organized into specific pages.
- Unstructured/free-text data, such as hospitalization reports, imaging reports, or discharge prescriptions.

Data from more than 20 sources are loaded daily into the Bordeaux University Hospital CDW. As of 2024, these data represent a total of (Table 1): 2,502,063 distinct patients; 20,982,497 inpatient or outpatient visits; and 3,474,264,570 observations, including 72,580,022 textual documents.

**Table 1:** Number of observations by source in the Bordeaux University Hospital clinical data warehouse.

Data source	Patients (n=2,502,063), n	Visits (n=20,982,497), n	Observations (n=3,474,264,570), n
Biology	1,286,459	4,157,738	1,323,818,025
Forms	1,641,829	8,130,391	829,328,415
Drug prescription/administration	744,611	1,775,078	469,279,982
Nursing care prescription/administration	688,450	1,875,347	263,813,687
Radiology	1,095,002	3,690,225	127,437,286
Localization	2,315,342	20,259,380	86,453,388
Free-text notes	2,045,379	12,202,309	72,580,022
Diagnostic (ICD-10^a^)	947,024	2,650,212	63,135,010
Care prescription/administration	725,741	1,582,745	54,581,222
Nurse notes	619,767	1,729,145	54,558,694
Microbiology	474,997	961,850	39,277,538
Demographic data	2,502,063	20,982,497	23,841,233
Procedure (CCAM^b^)	1,396,469	5,720,634	16,503,052
Chemotherapy	47,325	238,341	12,690,644
Nurse notes	733,021	2,143,927	12,002,868
Pathology	397,492	658,679	10,371,426
Surgery data	365,077	582,092	5,802,650
Other	435,078	748,627	4,112,204
Transfusion	217,389	491,076	2,647,208
Intensive care unit	8733	9349	2,030,016

^a^ICD-10: International Statistical Classification of Diseases and Related Health Problems, 10th Revision.

^b^CCAM: Classification Commune des Actes Médicaux (Common Classification of Medical Procedures; French terminology for coding procedures).

**Figure 2:**
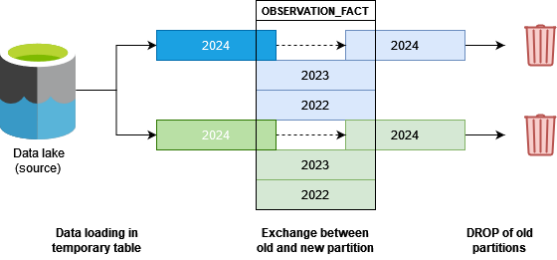
Partitioning strategy implemented at Bordeaux University Hospital on the OBSERVATION_FACT table. Data from various sources (represented by different colors) are extracted daily from the data lake and loaded into intermediate tables (dark colors). During this Extract, Transform, and Load (ETL) process, only a subset of source data—filtered based on the temporality of observations—is collected. Once all intermediate tables are ready, old partitions are successively replaced with new ones and then deleted. This approach ensures that only a portion of the data in the OBSERVATION_FACT table is reloaded, minimizing data downtime.

Data from the Bordeaux University Hospital CDW are stored in an Oracle RDBMS. Our implementation includes partitioning the OBSERVATION_FACT table by source and year (Figure 2). This partitioning strategy aims to facilitate daily data updates and optimize query performance on large data sets. As a result, over 700 partitions have been created. This setup allows data to be loaded from all or selected sources for 1 or multiple years. With this configuration, data for the current year are refreshed daily, while historical data are updated less frequently.

#### Elasticsearch

##### Elasticsearch: A Distributed NoSQL Database for Document Storage and Indexing

**Figure 3:**
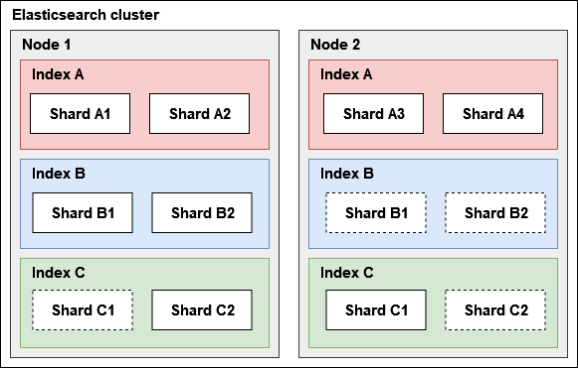
Elasticsearch organization and components. An Elasticsearch cluster consists of 2 nodes. Shards with a solid border represent primary shards, while those with a dotted border indicate replicated shards. Index A is divided into 4 primary shards, distributed across 2 nodes without replication. Index B has 2 primary shards stored on node 1 and 2 replicated shards stored on node 2. Index C is split into 2 primary shards, distributed across both nodes in the cluster.

Elasticsearch [38] is a distributed, document-oriented NoSQL database based on Apache Lucene (Apache Software Foundation) [44] indexing (Figure 3). It stores data as JSON documents in a schema-less structure. In Elasticsearch, an index (equivalent to a table in an RDBMS) stores documents, each of which corresponds to a tuple in an RDBMS table. These documents contain fields, which are equivalent to columns in an RDBMS table. The structure of an index is defined by mappings, which describe the fields in terms of column names, data types, and indexes—similar to how an RDBMS defines table structures. Each index consists of 1 or more shards, each corresponding to a Lucene inverted index [45], which is composed of 2 substructures:

- The dictionary of terms: A sorted list storing all terms included in the indexed documents.
- The postings list [46]: A structure that records, for each term, the list of document IDs containing the term, along with its position(s) within each document.

Queries in Elasticsearch are parallelized across multiple shards within an index, enhancing search performance. Primary shards can be replicated across different nodes within an Elasticsearch cluster (Figure 3), ensuring high availability. If a node goes offline, another available node with a replicated shard can still process queries, preventing downtime and ensuring continuity.

While NoSQL databases offer many advantages, systems such as Elasticsearch do not efficiently support join operations [47]. To enable querying similar to RDBMSs, a denormalization step is often required, involving data duplication to ensure all necessary information is contained within a single structure [47,48].

##### Elasticsearch Text Indexation

Elasticsearch supports various data types for fields, including text, which undergoes a specific indexing process using analyzers. This process builds a Lucene inverted index through 3 sequential steps:

- Character filtering: Cleans the text before indexing, such as removing special characters or replacing certain ones (eg, œ → oe).
- Tokenization: Splits the text into tokens. By default, Elasticsearch follows the Unicode Text Segmentation standard [49], which removes punctuation and uses spaces as separators.
- Token filtering: Modifies, removes, or adds tokens based on predefined rules. This step handles stop words, synonyms, and other text-processing enhancements.

The final output of this processing chain is stored in a Lucene inverted index.

##### Elasticsearch Keyword

The *keyword* data type is designed for structured data and can be used for both numerical and textual values. When a field is mapped as a keyword, it is stored in Lucene’s inverted index exactly as it is, without any preprocessing. For example, the text “BIO:ERYTHRO” will be indexed as “BIO:ERYTHRO” if typed as a keyword. However, if assigned a text data type, it will be split into separate entries: “bio” and “erythro” in the inverted index. Keyword fields are particularly efficient for aggregation, sorting, and exact-term searches.

### Adapting the i2b2 Data Model for Persistence in Elasticsearch

#### Overview

The adaptation of the i2b2 data model for persistence in Elasticsearch was carried out in 2 steps:

- Mapping the OBSERVATION_FACT table to an Elasticsearch index: This involved converting the data definition language used to create the OBSERVATION_FACT table into an equivalent observation_fact index structure, primarily defining field names, data types, and indexing modes.
- Modifying the observation_fact index structure by (1) incorporating elements from the patient and visit dimension tables, and (2) enriching observations with contextual information extracted from the ONT part of the i2b2 data model, stored as facets [50] of the observations. This modification was necessary due to Elasticsearch’s lack of support for traditional relational joins.

Finally, the persistence of the observation component of the i2b2 data model in Elasticsearch was evaluated.

#### Mapping the Native OBSERVATION_FACT Table Structure as the observation_fact Index

The first step involved mapping all the columns of the OBSERVATION_FACT table into an Elasticsearch index. In an Elasticsearch document, mappings define 2 main elements (Table 2):

- The data type of each field in the Elasticsearch index (eg, numeric types such as long or double, text, date, keyword).
- The indexing method for each field. For instance, if a field is assigned the text data type, the mapping specifies the analyzer(s) used to index its content.

Thus, the 2 criteria considered to map OBSERVATION_FACT were:

- The need to perform count or aggregation queries on the field. In this case, the Elasticsearch data type used for the field was keyword. In the OBSERVATION_FACT table, the fields used as aggregation criteria or in enumeration queries are fields corresponding to foreign keys in dimension tables:
  ENCOUNTER_NUM and PATIENT_NUM columns: These are used to count patients or visits, aggregate information by patient or by visit, etc;
  CONCEPT_CD, MODIFIER_CD, and PROVIDER_ID columns: These are used to count elements by clinical and biological criteria, aggregate information by provider, etc;
  INSTANCE_NUM column: This corresponds to the key for grouping a principal observation with its modified versions;
  TVAL_CHAR, VALUEFLAG_CD, and UNITS_CD columns: These are used in the case of numerical observations to specify elements concerning the numerical operator (=, >, ≥, etc), the interpretation in relation to limits, or the unit associated with the numerical result.
- The need to query a field with free-text queries: In this case, the text data type is associated with an analyzer for managing free-text. The column concerned in the OBSERVATION_FACT table corresponds to the OBSERVATION_BLOB column (in OBSERVATION_FACT, the TVAL_CHAR column is also used to store short text. In our implementation, all text [short or long] is stored in OBSERVATION_BLOB).

**Table 2:** Mapping of the native OBSERVATION_FACT columns to Elasticsearch types.

Column name	Column data type	Elasticsearch field data type	Analyzer
ENCOUNTER_NUM	NUMBER	Keyword	No
PATIENT_NUM	NUMBER	Keyword	No
CONCEPT_CD	VARCHAR2	Keyword	No
PROVIDER_ID	VARCHAR2	Keyword	No
START_DATE	DATE	Date	No
MODIFIER_CD	VARCHAR2	Keyword	No
INSTANCE_NUM	NUMBER	Keyword	No
VALTYPE_CD	VARCHAR2	Keyword	No
TVAL_CHAR	VARCHAR2	Keyword	No
NVAL_NUM	NUMBER	Double	No
VALUEFLAG_CD	VARCHAR2	Keyword	No
QUANTITY_NUM	NUMBER	Double	No
UNITS_CD	VARCHAR2	Keyword	No
END_DATE	DATE	Date	No
LOCATION_CD	VARCHAR2	Keyword	No
OBSERVATION_BLOB	TEXT	Text	Yes
CONFIDENCE_NUM	NUMBER	Double	No
UPDATE_DATE	DATE	Date	No
DOWNLOAD_DATE	DATE	Date	No
IMPORT_DATE	DATE	Date	No
SOURCESYSTEM_CD	VARCHAR2	Keyword	No
UPLOAD_ID	NUMBER	Long	No

#### Modification of the Structure of the observation_fact Index

**Figure 4:**
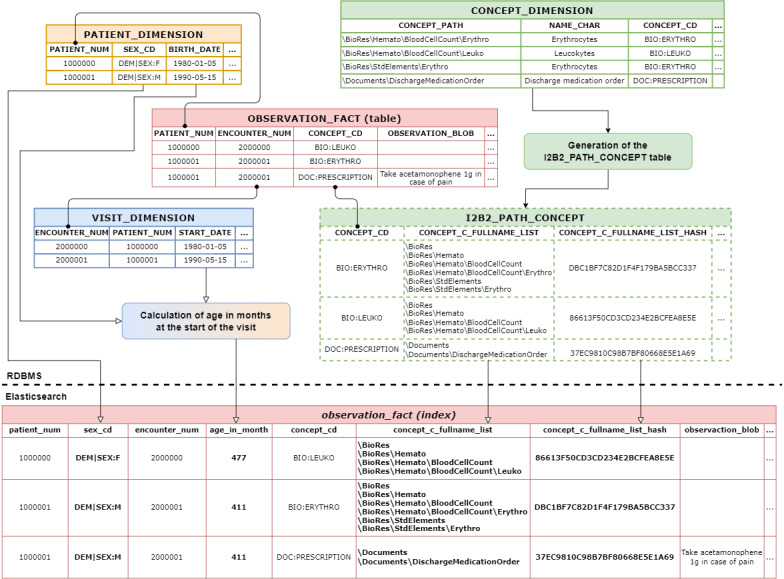
Creation of the observation_fact index from the different tables of the Informatics for Integrating Biology & the Bedside (i2b2) model. The table I2B2_PATH_CONCEPT (bordered with dotted line) is created based on CONCEPT_DIMENSION: for each CONCEPT_CD, the table contains the ordered list of CONCEPT_PATH, including all intermediate truncated versions of those paths. The algorithm used for the generation of the I2B2_PATH_CONCEPT table is provided in Multimedia Appendix 2. The relevant data from the PATIENT_DIMENSION, VISIT_DIMENSION, and I2B2_PATH_CONCEPT tables are added into the observation_fact index as a facet. BIRTH_DATE is used with visit START_DATE to compute the age_in_month_at_start_visit facet. The round-tipped arrows represent the joins between the tables. RDBMS: relational database management system.

As it is not possible to perform joins between indexes in Elasticsearch, 2 types of changes have been made to overcome this limitation (Figure 4):

- Some of the columns describing observations within the PATIENT_DIMENSION and VISIT_DIMENSION tables were added to the observation_fact index. For reasons of parsimony, only PATIENT_DIMENSION.SEX_CD, PATIENT_DIMENSION.BIRTH_DATE, and VISIT_DIMENSION.START_DATE have been integrated into the observation_fact index. PATIENT_DIMENSION.BIRTH_DATE and VISIT_DIMENSION.START_DATE have not been added as fields, but have been used to calculate the age_in_month_at_start_visit field;;
- To make documents of the observation_fact index accessible through the different hierarchical levels described in the ONT part, all the C_FULLNAME associated with the CONCEPT_PATH (also MODIFIER_PATH and PROVIDER_PATH) have been integrated into the observation_fact index. Additional tables have been added to the ONT part of the i2b2 model to allow the addition, by join, of all the paths (intermediate and final) for each possible value of the OBSERVATION_FACT lookup columns (ie, CONCEPT_CD, MODIFIER_CD, and PROVIDER_ID). The pseudo-code describing the generation of table I2B2_PATH_CONCEPT, for the CONCEPT_CD column, is available in Multimedia Appendix 2. Within the observation_fact index, paths are stored in 3 new fields called c_fullname_concept, c_fullname_modifier, and c_fullname_provider as lists of keywords. As a result, each observation within the observation_index is associated with all the paths (intermediate and full paths) describing all the lookup fields.

#### Evaluation of the Elasticsearch Persistence

The evaluation of the proposed implementation of the main part of the i2b2 data model in Elasticsearch aims to assess:

- The ability to load partial individual data (CRC part) daily. The specific mechanisms used for data loading are described. Loading performance is evaluated in terms of median loading time and the median number of documents loaded per minute.
- The ability to update metadata (ONT part). Similarly, the specific mechanisms implemented to update the metadata elements integrated into the observation_fact index are described. Metadata updating performance is evaluated in terms of median update time and the average number of concept_cd entries that need to be updated each week.

The ability to perform queries on the CDW persisted in Elasticsearch was evaluated. The results of count queries (distinct patients or distinct encounters) were compared between Oracle RDBMS and Elasticsearch implementations for the same CDW data in terms of result value and execution time (median execution time based on 20 executions). Different types of queries were performed, with criteria evaluated at the patient or encounter level (Table 3):

- Counting queries based on structured data (queries 1 and 2), including a query that returns a small number of patients (≈1500) and a query that returns a large number of patients (≈150,000).
- Counting queries based on structured data combined with a numerical filter (query 3).
- Counting queries based on free-text data (query 4).
- Counting queries based on structured data combined with a temporal filter (query 5).
- Combination of the above criteria in AND/OR Boolean queries, with multiple evaluation orders (eg, [(1) and (2)] vs [(2) and (1)]).

The hardware resources used for data persistence and query computation have been described.

**Table 3:** Query comparison between Oracle RDBMS^a^ and Elasticsearch.

Query/fields and query type					Query result type		Execution time, median (IQR)			Counts^b^, n
							Elasticsearch		Oracle RDBMS	
**(1) Pembrolizumab**										
	Nontemporal			Patients		0.22 (0.21-0.23)		0.15 (0.14-0.16)		1701
	Same encounter			Patients		0.43 (0.41-0.48)		0.18 (0.18-0.19)		1701
	Same encounter			Encounters		0.43 (0.41-0.49)		0.20 (0.19-0.20)		3986
**(2) Cancer**										
	Nontemporal			Patients		0.96 (0.95-0.98)		8.99 (8.66-9.51)		153,583
	Same encounter			Patients		4.81 (4.74-4.87)		16.07 (15.55-16.63)		153,583
	Same encounter			Encounters		4.79 (4.76-4.85)		15.97 (15.08-16.76)		516,682
**(3) Sodium ≥ 145 mmol/L**										
	Nontemporal			Patients		0.38 (0.36-0.41)		9.42 (8.16-10.05)		26,426
	Same encounter			Patients		0.75 (0.71-0.8)		9.39 (8.33-9.92)		26,426
	Same encounter			Encounters		0.77 (0.72-0.82)		9.41 (8.38-9.9)		33,070
**(4) Adenocarcinoma in free text**										
	Nontemporal			Patients		0.60 (0.59-0.62)		6.13 (5.97-6.51)		69,022/25,547
	Same encounter			Patients		3.27 (3.20-3.33)		7.27 (7.01-7.50)		69,022/25,547
	Same encounter			Encounters		3.27 (3.24-3.33)		7.26 (7.04-7.53)		369,953/92,638
**(5) Cancer in 2023**										
	Nontemporal			Patients		0.31 (0.29-0.32)		6.72 (6.53-7.25)		19,251
	Same encounter			Patients		0.66 (0.63-0.71)		6.88 (6.62-7.52)		19,251
	Same encounter			Encounters		0.66 (0.64-0.70)		7.03 (6.88-7.41)		3606
**(1) or (2)**										
	Nontemporal			Patients		1.00 (0.97-1.02)		9.15 (8.79-9.68)		153,589
	Same encounter			Patients		4.95 (4.91-5.02)		15.72 (15.41-16.39)		153,589
	Same encounter			Encounters		4.99 (4.91-5.05)		16.3 (15.74-16.81)		516,853
**(2) or (1)**										
	Nontemporal			Patients		0.99 (0.97-1.02)		9.04 (8.79-9.32)		153,589
	Same encounter			Patients		4.96 (4.89-5.02)		15.85 (15.38-16.76)		153,589
	Same encounter			Encounters		4.95 (4.91-5.04)		16.51 (15.44-16.91)		516,853
**(1) and (2)**										
	Nontemporal			Patients		1.07 (1.04-1.10)		6.68 ((6.45-6.95)		1695
	Same encounter			Patients		4.81 (4.77-4.90)		6.83 (6.52-7.33)		1671
	Same encounter			Encounters		4.81 (4.74-4.91)		6.94 (6.61-7.12)		3815
**(2) and (1)**										
	Nontemporal			Patients		1.09 (1.06-1.10)		10.49 (10.17-11.12)		1695
	Same encounter			Patients		4.96 (4.85-5.02)		21.38 (20.46-22.62)		1671
	Same encounter			Encounters		4.94 (4.89-5.01)		21.38 (20.75-22.08)		3815
**(5) and (3)**										
	Nontemporal			Patients		0.64 (0.59-0.69)		15.54 (14.41-17.51)		1917
	Same encounter			Patients		1.45 (1.37-1.51)		16.15 (14.96-18.42)		1005
	Same encounter			Encounters		1.47 (1.4-1.57)		16.42 (14.95-18.05)		1135
**(1) and (5) and (3)**										
	Nontemporal			Patients		0.89 (0.82-0.93)		15.68 (14.8-17.29)		78
	Same encounter			Patients		2.05 (1.85-2.08)		15.76 (14.59-17.98)		11
	Same encounter			Encounters		1.95 (1.91-2.12)		15.61 (13.84-17.23)		11

^a^RDBMS: relational database management system.

^b^If Oracle RDBMS and Elasticsearch return the same result, a single count is provided. Otherwise, the count obtained with Elasticsearch is presented first, followed by the count obtained with Oracle RDBMS.

## Results

### Elasticsearch Data Loading

A specific ETL process was developed using Spring Boot [51] to convert data from an RDBMS into Elasticsearch.

Data from PATIENT_DIMENSION and VISIT_DIMENSION were added to the OBSERVATION_FACT table using outer joins in the ETL. For the ONT data, intermediate tables were created to store the ordered list of all CONCEPT_PATH (MODIFIER_PATH and PROVIDER_PATH, respectively) for each CONCEPT_CD (MODIFIER_CD and PROVIDER_ID, respectively). An example for the CONCEPT_CD column is presented in Table 4.

As mentioned above, the data in our OBSERVATION_FACT table are partitioned by data source and year. A similar rolling strategy has been implemented in Elasticsearch, as presented in Multimedia Appendix 3. Consequently, observation_fact consists of multiple indexes, corresponding to the partitions in OBSERVATION_FACT. Each observation_fact index was divided into 3 primary shards (1 per node) to enable parallel queries, with no replicated shards created.

All data from the OBSERVATION_FACT table were loaded into an Elasticsearch cluster, comprising 2,502,063 patients, 20,982,497 visits, and 3,474,264,570 observations. The number of records in the observation_fact indexes matches that in the OBSERVATION_FACT table. Data persistence in Oracle RDBMS requires 1.030 TB for data and 2.640 TB for indexes (including 420 GB for the Oracle RDBMS full-text index). By contrast, data persistence in Elasticsearch requires only 1.635 TB, representing 44% of the disk space needed for Oracle RDBMS.

**Table 4:** The i2b2^a^ PATH_CONCEPT table: a generated metadata table that lists all available C_FULLNAME paths for each CONCEPT_CD described in the ONT^b^. Equivalent tables exist for MODIFIER_CD and PROVIDER_ID.

CONCEPT_CD	CONCEPT_C_FULLNAME_LIST	CONCEPT_C_FULLNAME_LIST_HASH
BIO:ERYTHRO	\BioRes \BioRes\Hemato \BioRes\Hemato\BloodCellCount \BioRes\Hemato\BloodCellCount\Erythro \BioRes\StdElements \BioRes\StdElements\Erythro	DBC1BF7C82D1F4F179BA5BCC337
BIO:LEUKO	\BioRes \BioRes\Hemato \BioRes\Hemato\BloodCellCount \BioRes\Hemato\BloodCellCount\Leuko	86613F50CD3CD234E2BCFEA8E5E
DOC:PRESCRIPTION	\Documents \Documents\DischargeMedicationOrder	37EC9810C98B7BF80668E5E1A69

^a^i2b2: Informatics for Integrating Biology & the Bedside.

^b^ONT: ontology management cell.

The duration of data loading in Elasticsearch by source is provided in Table 5. For the full year 2023 (approximately 340,000,000 observations), the median overall data loading time with 10 sources in parallel is 7.41 hours, corresponding to a median indexing rate of 46,546,000 documents per hour. Indexing speed appears to depend on the type of data, with structured numerical data being indexed at a median rate of 171,600 documents per minute—approximately 4 times faster than free-text data, which has a median rate of 48,100 documents per minute. Data loading in Elasticsearch has not been compared with data loading in Oracle DBMS. Indeed, data loading in Oracle RDBMS is heavily influenced by the transformations performed when integrating data from the source information system into the OBSERVATION_FACT table. By contrast, as loading into Elasticsearch is conducted from the OBSERVATION_FACT table, only transformations related to data denormalization are applied during the Elasticsearch loading process.

**Table 5:** Data loading metrics for the full year 2023 (metrics based on 8 loads).

Source	Observations (n=339,331,147), n	Documents (×1000/minute), median (Q1-Q3)^a^	Loading duration (minutes), median (Q1-Q3)^b^^,c^
Chemotherapy	478,438	185.3 (132.1-222.8)	2.5 (2.1-4.9)
Forms	77,460,099	181.6 (164.4-188.9)	429.1 (407.3-490.1)
Biology	158,384,678	171.6 (162.4-176.5)	916.4 (893.1-968.3)
Drug prescription/administration	32,736,762	155.6 (148.6-199.3)	204.5 (187.4-214.6)
Nursing care prescription/administration	16,523,635	107.4 (87.2-138.8)	149.2 (113.7-180.2)
Radiology	9,438,970	76.6 (37.8-91.7)	130.8 (79.9-201.4)
Diagnostic (ICD-10^d^)	6,690,589	64.6 (57.3-105.7)	105.2 (64.2-116.8)
Localization	6,181,496	59.8 (54.3-79.2)	102.8 (77.6-118.3)
Demographic data	2,142,053	59.6 (55.0-122.6)	34.7 (17.3-37.8)
Nurse notes	12,984,197	58.8 (52.4-66.7)	223.6 (199.0-248.7)
Care prescription/administration	4,930,320	56.5 (30.0-68.6)	86.9 (71.7-162.6)
Free-text notes	6,066,806	48.1 (39.8-78.3)	125.9 (80.2-151.3)
Surgery data	518,998	13.9 (6.5-248.9)	37.4 (2.1-80.3)
Microbiology	3,364,431	13.2 (11.5-17.6)	254.3 (189.7-290.2)
Pathology	678,289	10.7 (7.6-18.8)	65.6 (38.8-89.4)
Other	284,220	4.9 (3.1-190.7)	59.1 (1.5-90.6)
Transfusion	467,166	2.7 (2.1-3.1)	165.3 (152.4-205.4)

^a^Overall: 775.8 (749.2-792.1).

^b^The median loading duration was calculated without considering parallelization. Some sources consist of multiple indexes, such as biology (which comprises 5 indexes) and forms (which comprises 4 indexes). This explains why the maximum median loading duration (916.4 minutes) is higher than the overall median duration (444.8 minutes).

^c^Overall: 444.8 (429.0-461.2).

^d^ICD-10: International Statistical Classification of Diseases and Related Health Problems, 10th Revision.

### Elasticsearch Metadata Updating

In the case of a modification in the ONT part, a strategy for updating the observation_fact indexes has been implemented. This strategy is presented and evaluated in Multimedia Appendix 4 [52].

### Elasticsearch Querying

The results of queries executed on an Oracle RDBMS engine were compared with those obtained using Elasticsearch. For queries involving joins (AND Boolean queries), the joins were performed at the application layer for both Oracle RDBMS and Elasticsearch. A full-text index was available in the Oracle RDBMS. All necessary indexes for the Oracle RDBMS queries were calculated, and the statistics were up to date. The results and response times were compared between the 2 engines. The Oracle RDBMS (version 19.17) runs as a cluster on 2 servers, each with 80 CPUs and 1 TB of memory. The Elasticsearch database (version 7.17.3) is configured as a 3-node cluster, deployed through Docker, with each node having access to 25 CPUs and 30 GB of memory.

A total of 9 different queries were compared (Table 3). Each query evaluated criteria either at the patient level (nontemporal queries) or at the visit level (temporal queries). To assess execution time, each query was executed 20 times on both engines. The results were identical between the Oracle RDBMS engine and Elasticsearch, except for the full-text query, which returned more results in Elasticsearch due to differences in full-text indexing implementation. Response times were generally lower with Elasticsearch than with Oracle RDBMS, except for the “Pembrolizumab” query, which returned a low number of results. Temporal queries (evaluating criteria within the same encounter) were consistently slower than nontemporal queries on both engines.

### Source Code

The ETL between i2b2 RDBMS and i2b2 Elasticsearch was developed using Spring Boot (a Java framework; VMware). The source code of the ETL is available at [53].

## Discussion

### Principal Findings

To our knowledge, this work is the first to evaluate the technical feasibility and performance of persisting an i2b2 model in an Elasticsearch database. Beyond demonstrating feasibility, the evaluation highlights significant improvements in overall query times and hardware resource requirements, particularly in storage, with a 66% reduction in disk space usage. Additionally, the implementation has been tested on a large volume of data, demonstrating good scalability. The proposed implementation is currently in production at Bordeaux University Hospital, supporting more than 200 research projects since 2022.

Elasticsearch is designed for very fast searches, prioritizing optimization for speed, high availability, and horizontal scalability. However, this comes at the cost of certain transactional guarantees (ACID) and absolute precision in some operations, such as counts. Specifically, in the event of a failure during the update of a replicated index, the system may restart in an inconsistent state, where data are updated in the primary shard but not across all its replicas. In our context, high availability is not a major concern. We have therefore chosen not to replicate shards across different cluster nodes, avoiding consistency issues in case of failure. Additionally, in the rolling strategy implemented for data loading, a newly created index becomes available for searching only after it has been fully loaded, minimizing the risk of querying incomplete data. To optimize queries, Elasticsearch employs probabilistic counting algorithms (eg, HyperLogLog) for count queries. However, in our context, exact results are essential for this type of query. We have therefore configured Elasticsearch to ensure accuracy, sacrificing some query performance. Despite this, execution times were consistently lower with Elasticsearch than with the RDBMS.

In our implementation, we chose to add the lookup columns from the dimension tables and the paths of CONCEPT_CD (as well as MODIFIER_CD and PROVIDER_ID) as facets of the observation_fact indexes. Combined with fields of the keyword data type, this approach optimizes query times, particularly for enumeration queries, at the expense of indexing time, which is not performed in real time. The disadvantage of this implementation is that data (CRC) and metadata (ONT) are not kept separate, meaning that any modification to the metadata requires reloading or updating the data. To address this issue, we implemented a process for updating metadata within the observation_fact indexes and demonstrated the feasibility of performing these updates without reloading the entire data set.

An alternative implementation would involve maintaining the observation_fact indexes without adding additional fields and instead creating separate indexes for the various dimension tables and the ONT metadata table. Similar to the proposed configuration, joins at the application layer (ie, intersecting patient or encounter lists) would be used to combine results from different indexes for AND Boolean queries. However, this approach presents potential performance challenges. For instance, intersecting gender data (eg, female) stored in the patient_dimension index with a criterion stored in the observation_fact indexes would require loading into memory the list of all patient keys corresponding to women in the CDW—approximately 1.2 million patients in the Bordeaux University Hospital implementation. This issue becomes even more pronounced when handling data from the VISIT_DIMENSION table.

Alternatively, an Elasticsearch implementation could have used nested field data types [54]. These types allow the content of a nested field to be indexed as a separate Lucene document within the main document. This approach would have enabled indexing a patient document containing visit documents as nested data, which in turn would contain observation documents as nested data. However, this implementation would have required reloading all data for a given patient at once, which was incompatible with our goal of reloading recent data daily. Additionally, queries involving nested documents generally perform worse than standard field queries.

In our implementation, we mapped the TVAL_CHAR column of the OBSERVATION_FACT table as a “keyword” in the indexes constituting observation_fact. In the i2b2 data model, the TVAL_CHAR column is used in 2 cases, in conjunction with the content of the VALTYPE_CD column:

- VALTYPE_CD = “T”: The TVAL_CHAR column contains the short text associated with the CONCEPT_CD (eg, a biology comment).
- VALTYPE_CD = “N”: The TVAL_CHAR column contains the mathematical operator associated with the numerical value in the NVAL_NUM column (eg, “E” for “equal,” “NE” for “not equal,” and “L” for “less than”).

In the i2b2 implementation at Bordeaux University Hospital, all free-text data, including short texts, are stored in the OBSERVATION_BLOB column. As a result, the TVAL_CHAR column contains only the mathematical operators for numerical values. In a fully i2b2-compliant implementation, it would be necessary not only to map the TVAL_CHAR column as a keyword but also to associate an analyzer with it. This would enable both full-text search and aggregate queries.

To evaluate query capabilities using the Elasticsearch database, part of the i2b2 query engine was reimplemented. This reimplementation supports multicriteria enumeration queries (ie, “AND,” “OR,” and “NOT” Boolean queries), including numerical data and free-text search, evaluated at the patient or encounter level. However, not all functionalities available in the original i2b2 query engine have been implemented. In particular, the ability to perform queries based on the number of occurrences of observations (eg, identifying patients who have visited the hospital at least three times) or queries that account for temporality between events (eg, patients with hemoglobin measurements within 5 days after colon surgery) has not been implemented. These 2 types of queries are more complex than those already implemented, as they require additional observation aggregation and aggregate filtering steps before results can be aggregated at the patient or event level. However, the organization of the observation_fact indexes proposed here appears to be compatible with executing such queries. Implementing these capabilities could be the focus of future work.

The proposed ETL for loading the Elasticsearch database is based on data already integrated into an i2b2 model persisted in an RDBMS. Beyond enabling a straightforward ETL process from i2b2 RDBMS to i2b2 Elasticsearch, maintaining this relational database appears essential for specific use cases that would not be feasible with Elasticsearch alone (eg, joins with the system containing the source data, preparation of complex data by joining OBSERVATION_FACT to itself). Consequently, while Elasticsearch provides performance improvements, these must be weighed against the requirement to maintain 2 parallel infrastructures.

Although no i2b2 implementation using a NoSQL database has been described to our knowledge, the literature includes examples of CDWs designed for free-text analysis. OpenMRS [55], an open-source EHR project, features a free-text search module based on Apache Lucene, integrated with Hibernate Search. R-oogle [20], a document-oriented CDW, preindexes free-text data using external terminologies (eg, Medical Subject Headings [MeSH]) and stores them in a Lucene index. Dr. Warehouse [21,56] implements a segmentation approach for free-text documents, breaking them into “propositions” stored in a full-text index within the Oracle RDBMS. These propositions are linked to contextual elements (eg, antecedents, personal and family history, or negation). Doc’EDS [57] provides a phenotyping search engine that integrates both structured and unstructured data, indexed through Apache Lucene. All CDW implementations utilizing NoSQL databases [20,55,57] rely on Apache Lucene’s low-level libraries. Elasticsearch, built on Apache Lucene, operates at a higher abstraction level, simplifying deployment and integration. While this limits fine-tuning capabilities for specific indexing needs, it provides significant advantages, such as access to the Kibana [58] visualization engine and query clients available in Java via Spring Boot [59].

### Conclusions

This study presents an Elasticsearch-based implementation of the i2b2 data model for phenotyping tasks. By reimplementing part of the i2b2 search engine, the study demonstrates that Elasticsearch provides similar query capabilities while offering faster performance and improved efficiency for free-text searches.

## Appendix

Multimedia Appendix 1 Workflow for using data contained in an Informatics for Integrating Biology & the Bedside clinical data warehouse.

Multimedia Appendix 2 Pseudocode of the I2B2_PATH_CONCEPT table construction. i2b2: Informatics for Integrating Biology & the Bedside.

Multimedia Appendix 3 Rolling strategy for data loading.

Multimedia Appendix 4 Elasticsearch metadata update strategy.

## Abbreviations

ACID: atomicity, consistency, isolation, and durability
CDW: clinical data warehouse
CRC: clinical research chart
EHR: electronic health record
ETL: Extract, Transform, and Load
i2b2: Informatics for Integrating Biology & the Bedside
ICD-10: International Statistical Classification of Diseases and Related Health Problems, 10th Revision
LOINC: Logical Observation Identifiers Names and Codes
MeSH: Medical Subject Headings
OMOP-CDM: Observational Medical Outcomes Partnership-Common Data Model
ONT: ontology management cell
RDBMS: relational database management system
