# Supplementary material for: Integrating Health Care Data in an Informatics for Integrating Biology & the Bedside (i2b2) Model Persisted Through Elasticsearch: Design, Implementation, and Evaluation in a French University Hospital
Source: JMIR Med Inform. 2025 Apr 24;13:e65753. doi: 10.2196/65753 (PMC12062766; doi:10.2196/65753)
Supplement: Multimedia Appendix 1 [file medinform_v13i1e65753_app1.docx]

### Appendix 1

This section describes the workflow for using data contained in an i2b2 CDW to identify patients eligible for inclusion in a research project (phenotyping). The aim is to identify a list of patients based on several criteria (diagnoses, value of biological results, presence of a specific word in a clinical notes, etc.).

The i2b2 platform provides a query engine for this use case, called “i2b2 web client” (Figure 5). The query tool is made up of two parts:

- A part corresponding to a hierarchical organization of the data dictionaries integrated into i2b2 (called “metadata”). Metadata is organized as a multi-hierarchical tree. Querying a node in the hierarchy corresponds to querying all the descendant leaf nodes (direct or indirect) from the current (e.g. query ‘C30-C39’ node from ICD-10 integrated into i2b2 is equivalent to querying all the observations attached to the ICD-10 codes ‘C30.0’, ‘C30.1’, etc.).
- A part for building phenotyping queries on the i2b2 CDW through panels. Nodes available in the metadata part can be drag-and-dropped inside the panels. It is also possible to apply search criteria at node level (temporal filter, numerical value, textual value, etc). Queries are composed using different panels:
  - Within a panel, the different criteria are evaluated using the Boolean operator ‘OR’;
  - Different panels are evaluated using the Boolean operator ‘AND’.

Once the query has been constructed, it can be executed, to obtain the number of patients corresponding to the criteria and generate the list of patients corresponding to the query. Once the patients have been identified, the data relating to them contained in the CDW is made available to the researchers.

The Figure 5 provides an example of usage of i2b2 query tool. The aim of this query is to identify male patients with adenocarcinoma of the lung:

- Panel 1: correspond to patients with a diagnosis of lung cancer coded in ICD-10 (in the OBSERVATION_FACT table);
- Panel 2: correspond to patients with the term ‘adenocarcinoma’ in some reports (in the OBSERVATION_FACT table);
- Panel 3: correspond to patients with male gender (in the PATIENT_DIMENSION table).

Panels are evaluated using the Boolean operator ‘AND’.


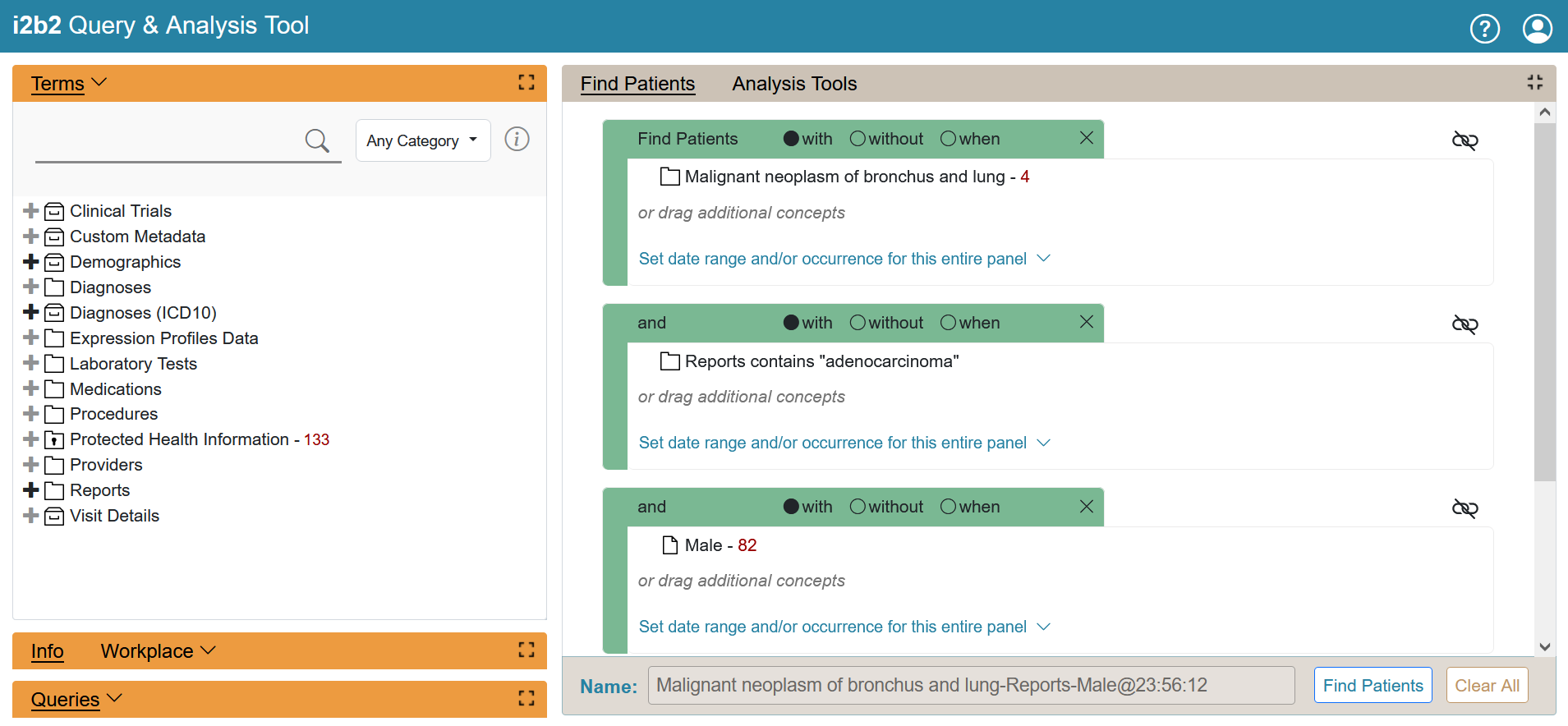
Figure 5. Example of a query to identify patients with lung adenocarcinoma.

In terms of SQL queries, the i2b2 search engine intersects the results of three different queries :

1. Panel 1: patients with a diagnosis of lung cancer coded in ICD-10. The CONCEPT_PATH (‘\ICD10\Diagnoses\C00-D49\C00-C96\C00-C75\C30-C39\C34’) in the SQL sub-query corresponds to the C_FULLNAME of the “Malignant neoplasm of bronchus and lung” node of the ONT part. All the CONCEPT_CDs of the selected node's children in the ONT are obtained using “LIKE”.

SELECT DISTINCT PATIENT_NUM

FROM OBSERVATION_FACT

WHERE CONCEPT_CD IN (

SELECT CONCEPT_CD

FROM CONCEPT_DIMENSION

WHERE CONCEPT_PATH LIKE ‘\ICD10\Diagnoses\C00-D49\C00-C96\C00-C75\C30-C39\C34%’

)

1. Panel 2: patients with the term ‘adenocarcinoma’ in some reports. The CONCEPT_PATH (‘\i2b2\Reports\’) in the SQL sub-query corresponds to the C_FULLNAME of the “Reports” node of the ONT part. All the CONCEPT_CDs of the selected node's children in the ONT are obtained using “LIKE”. The term 'adenocarcinoma' is searched in the OBSERVATION_BLOB field of the OBSERVATION_FACT table.

SELECT DISTINCT PATIENT_NUM

FROM OBSERVATION_FACT

WHERE CONCEPT_CD IN (

SELECT CONCEPT_CD

FROM CONCEPT_DIMENSION

WHERE CONCEPT_PATH LIKE ‘\i2b2\Reports\%’

)

AND OBSERVATION_BLOB LIKE ‘%adenocarcinoma%’

1. Panel 3: patients with male gender. The node "Male" in the ONT refers to a specific value (‘DEM|SEX:M’) in the SEX_CD column of the PATIENT_DIMENSION table.

SELECT DISTINCT PATIENT_NUM

FROM PATIENT_DIMENSION

WHERE SEX_CD = ‘DEM|SEX:M’
