# Supplementary figures and images for: Integrating Health Care Data in an Informatics for Integrating Biology & the Bedside (i2b2) Model Persisted Through Elasticsearch: Design, Implementation, and Evaluation in a French University Hospital
Source: JMIR Med Inform. 2025 Apr 24;13:e65753. doi: 10.2196/65753 (PMC12062766; doi:10.2196/65753)

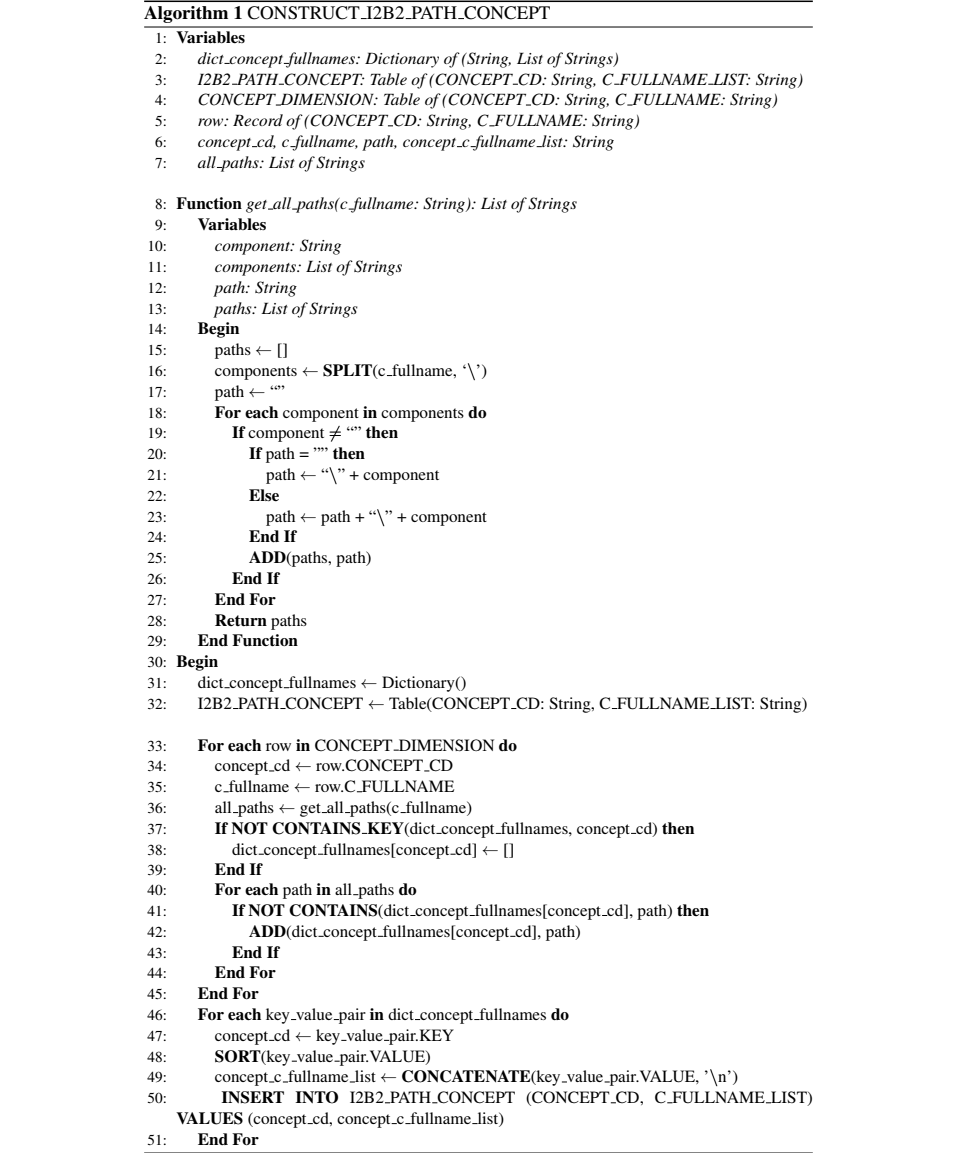

Supplement: Multimedia Appendix 2 [file medinform_v13i1e65753_app2.png]
