# Supplementary material for: Integrating Health Care Data in an Informatics for Integrating Biology & the Bedside (i2b2) Model Persisted Through Elasticsearch: Design, Implementation, and Evaluation in a French University Hospital
Source: JMIR Med Inform. 2025 Apr 24;13:e65753. doi: 10.2196/65753 (PMC12062766; doi:10.2196/65753)
Supplement: Multimedia Appendix 3 [file medinform_v13i1e65753_app3.docx]

### Appendix 3

Rolling strategy for data loading

Data contained in our OBSERVATION FACT table is partitioned by data source and by year. A similar organization has been implemented for Elasticsearch persistence, with the creation of an index for each partition of the source table. To ensure that the index structure is the same for each partition, all the indexes have been created according to an Elasticsearch index-template^[[1]](#footnote-1)^. To limit the time during which observations are not accessible to queries, the following rolling strategy has been implemented for data loading:

1. Creation of the new index based on the observation index-template. The index name is defined using the following convention: observation‑ [source‑name]-[year]-[index‑creation‑timestamp]. The [index‑creation timestamp] part is used to ensure the uniqueness of the index within the Elasticsearch database;
2. Data loading into the new index using the Elasticsearch bulk API, by batch of 500 observations;
3. Deletion of existing indexes for which an observation‑[source‑name]‑[year] alias^[[2]](#footnote-2)^ was found;
4. Addition of two aliases to the newly created index:
   1. The observation‑[source‑name]‑[year] alias, which is used to find the index to delete the next time the data source is loaded for a specific year;
   2. The observation alias, which is identical for all observation fact indexes. The observation alias is used as a source in Elasticsearch queries, allowing us to query all sources from all years transversely.

1. An index-template contains the structure definition of an Elasticsearch index. It can be used to define the structure of a new index at creation time (equivalent to inheritance). [↑](#footnote-ref-1)
2. In Elasticsearch, an alias is used to give an alternative name to an index. In our case, the alias used corresponds to the name of the index without the time-stamped part corresponding to the creation date of the index, which enables us to find the existing indexes corresponding to the source for a specific year being loaded. [↑](#footnote-ref-2)
