# Supplementary material for: Integrating Health Care Data in an Informatics for Integrating Biology & the Bedside (i2b2) Model Persisted Through Elasticsearch: Design, Implementation, and Evaluation in a French University Hospital
Source: JMIR Med Inform. 2025 Apr 24;13:e65753. doi: 10.2196/65753 (PMC12062766; doi:10.2196/65753)
Supplement: Multimedia Appendix 4 [file medinform_v13i1e65753_app4.docx]

### Appendix 4

Elasticsearch metadata update strategy

Due to the impossibility of performing joins in Elasticsearch, all the access paths for the various lookup dimensions have been integrated into the observation fact indexes in the form of a list of keywords (facets). A strategy for updating metadata, aimed at not reloading all the data, has been put in place to take account of ONT updates. The update strategy for the concept_c_fullname_list is as follows:

1. At load time, in addition to storing the list of c_fullnames attached to each concept cd in the concept_c_fullname_list field, we also store a hash of this ordered list in the concept_c_fullname_list_hash field (last column of Table 3).
2. During the metadata update process, for each index:
   1. We first generate the distinct list of tuples (concept_cd; concept_c_fullname_hash) available in each observation fact index, including concept cd for which the concept_c_fullname_hash is empty (i.e. cases where a CONCEPT_CD is not described in the ONT).
   2. We then query the ONT to get access to all triples (CONCEPT_CD; CONCEPT_C_FULLNAME_LIST; CONCEPT_C_FULLNAME_LIST_HASH). It is then possible to identify all the concept cd that need to be updated by identifying those for which the available hash is not the same as that obtained from the ONT.
   3. For each concept cd within each observation fact index, we finally update all the documents using Elasticsearch update by query and scripting[48]. This Elasticsearch functionality allows to update documents that match a specific query:
      1. The search component allows the update to target specific documents that need it (those whose concept cd has been identified as requiring update).
      2. The script component allows the value of a field to be replaced by another value. Here, we replace the old concept_c_fullname_list with the newly updated list, as well as the concept_c_fullname_hash of this ordered list.

Metadata added in observation fact indexes is updated every week in the CDW. The average number of CONCEPT CD requiring updating each week is 9,324 corresponding to 51,904,783 observations. For this process, the median update time is 119.5 minutes (IQR [74.5; 146.3]).
